# Climate-Driven Variation in Yellowfin Tuna Productivity in the Western and Central Pacific Ocean Inferred from a State-Space Model

**DOI:** 10.3390/ani16050856

**Published:** 2026-03-09

**Authors:** Xiaodong Li, Zhe Geng, Jie Cao, Jizhang Zhu, Jiangfeng Zhu

**Affiliations:** 1 College of Marine Living Resource Sciences and Management, Shanghai Ocean University, Shanghai 201306, China; 2 Key Laboratory of Sustainable Exploitation of Oceanic Fisheries Resources, Ministry of Education, Shanghai 201306, China; 3 Key Laboratory of Oceanic Fisheries Exploration, Ministry of Agriculture and Rural Affairs, Shanghai 201306, China; 4 National Engineering Research Centre for Oceanic Fisheries, Shanghai Ocean University, Shanghai 201306, China; 5 Department of Applied Ecology, Center for Marine Sciences and Technology, North Carolina State University, Morehead City, NC 28557, USA

**Keywords:** yellowfin tuna, time-varying productivity, state-space model, Pacific Decadal Oscillation, mixed layer thickness, ecosystem-based fisheries management

## Abstract

Understanding temporal variation in population productivity is critical for effective assessment and management of pelagic fish stocks under a changing climate. In this study, we applied a stochastic surplus production model in continuous time (SPiCT) with time-varying parameters to evaluate the productivity dynamics of yellowfin tuna (*Thunnus albacares*) in the western and central Pacific Ocean and to examine the influence of environmental variability on productivity. Multiple time-varying parameterization scenarios were explored to characterize uncertainties in productivity estimates and associated biological reference points. Generalized additive models were subsequently used to quantify the relationships between environmental variables and time-varying productivity. Results indicate that productivity estimates exhibit consistent temporal patterns across alternative modeling scenarios, while their magnitude and associated uncertainty are sensitive to model structure. Among the environmental factors examined, the Pacific Decadal Oscillation (PDO) and mixed layer thickness (MLT) showed consistent and statistically significant associations with maximum net productivity. Higher PDO values and greater MLT were both positively associated with population productivity. Overall, the results highlight the importance of environmental variability in shaping time-varying productivity of yellowfin tuna and demonstrate the feasibility of incorporating key environmental indicators into a state-space model. This approach provides a complementary framework for interpreting stock dynamics and supports the development of ecosystem-based fisheries management strategies in the western and central Pacific.

## Simple Summary

Climate change is affecting ocean conditions and the productivity of pelagic fish populations, creating challenges for fisheries assessment and management. Yellowfin tuna (*Thunnus albacares*) is an important species in the western and central Pacific Ocean, where productivity varies over time in response to environmental variability. In this study, we applied a population model that allows productivity to change through time to examine the productivity dynamics of yellowfin tuna and their relationship with environmental variability. Different modeling scenarios were compared to evaluate uncertainties in productivity estimates and management reference points. We found that while temporal patterns of productivity were generally consistent, their magnitude and associated uncertainty depended on model structure. In addition, large-scale climate variability and ocean conditions, particularly the Pacific Decadal Oscillation and mixed layer thickness, were positively associated with tuna productivity. These results emphasize the importance of incorporating environmental information into fisheries assessments to support ecosystem-based management under climate change.

## 1. Introduction

Accurately characterizing fish population dynamics under changing environmental conditions remains a fundamental challenge in fisheries stock assessment [1]. Traditional stock assessment models typically assume temporally constant parameters, such as the intrinsic growth rate (*r*) and carrying capacity (*K*). This assumption implies that fishing pressure is the primary driver of population dynamics and productivity, while largely neglecting other potential drivers such as environmental variability [2,3]. However, a growing body of evidence indicates that these parameters are not static but instead vary over time in response to climatic fluctuations, habitat modifications, and ecological interactions [4,5,6]. Failure to account for such temporal variability may result in biased estimates of population status and ultimately undermine the scientific robustness and reliability of fisheries management advice.

Some studies have investigated environmental and climatic influences on fish populations; however, their integration into stock assessment models through time-varying biological parameters remains limited. In data-rich, age-structured assessment models, climate effects can be incorporated by allowing key biological processes—such as recruitment, growth, maturation, or natural mortality—to vary with environmental conditions, thereby explicitly linking climate variability to population dynamics [7]. In contrast, data-moderate frameworks, typified by surplus production models, generally lack well-established methods for representing climate impacts through changes in biological parameters, which limits their ability to capture environmentally driven productivity shifts. In many existing studies, time-varying productivity is therefore incorporated by directly introducing selected environmental variables as covariates into assessment models, most commonly to explain variability in recruitment and growth processes [8,9,10]. This approach is often constrained by the limited availability and selection of environmental covariates, as well as uncertainties associated with missing data and model specification [11]. Moreover, changes in fish population productivity are widely recognized as the result of complex interactions among multiple abiotic and biotic drivers, rather than being attributable to a small number of predefined environmental factors [12]. This highlights the need for alternative approaches that can represent climate effects through dynamic biological parameters, particularly in data-moderate models such as surplus production frameworks.

Recent advancements in state-space and surplus production modeling have enabled the incorporation of time-varying parameters, providing a more flexible framework for capturing environmentally driven variation in population productivity [13,14]. Allowing key parameters such as *r* and *K* to vary over time can better represent how altered recruitment patterns, variable prey availability, and episodic environmental events affect fish populations, thereby reflecting the non-stationary nature of biological processes and management reference points [12,15,16]. Because Maximum Sustainable Yield (*MSY*) and its associated reference points are direct functions of *r* and *K*, temporal variability in these parameters can lead to substantial temporal shifts in MSY-based management targets [17]. In contrast to covariate-driven formulations, stochastic state-space approaches allow key population parameters to evolve dynamically through time without requiring environmental drivers to be specified a priori [13,14]. Moreover, while many previous studies have examined temporal variation in a single productivity parameter (e.g., *r* or *K* in isolation), allowing multiple productivity parameters to vary simultaneously may provide a more realistic representation of population dynamics and associated reference points. Quantifying how environmental drivers influence time-varying productivity and management benchmarks is therefore essential for developing adaptive and precautionary harvest strategies, particularly for stocks exposed to pronounced environmental variability.

In this study, we aim to address two key scientific questions regarding climate-driven variation in fish population productivity. First, using yellowfin tuna in the western and central Pacific Ocean as the study species, we evaluate whether stock status, biological reference points, and their associated uncertainties differ systematically between state-space surplus production models with time-varying parameters and traditional formulations assuming constant productivity. Second, we examine whether the process variation estimated from fisheries data, which represents unexplained deviations in population dynamics, is associated with environmental variability, and thus reflects potential climate signals in model residual dynamics. To address these questions, we fit a state-space surplus production model using SPiCT and formulate several time-varying scenarios in which *r* and *K* evolve dynamically over time. We then evaluate how these dynamics influence MSY-based biological reference points. Finally, generalized additive models (GAMs) are used to test whether environmental variables are associated with the estimated time-varying parameters and the model-derived process variation. By jointly allowing multiple productivity parameters to vary dynamically and linking both parameter evolution and process variation to environmental drivers, this study provides a flexible framework for detecting climate-driven productivity changes within data-moderate surplus production models. The results offer practical implications for fisheries assessment by improving the realism and uncertainty characterization of MSY-based reference points, and support the development of more adaptive and precautionary management strategies under increasing environmental variability.

## 2. Materials and Methods

### 2.1. Fisheries Data

**Figure 1:**
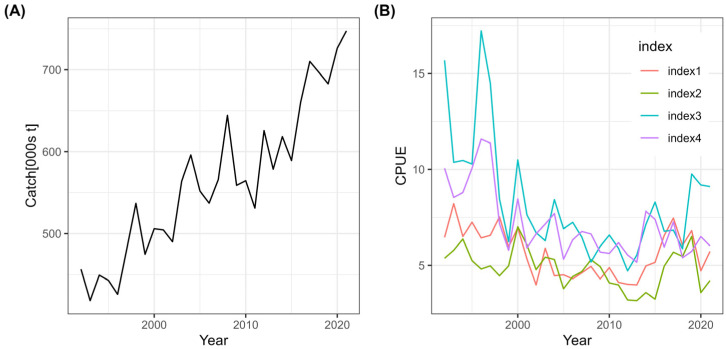
Yellowfin tuna catch and CPUE data in the central and western Pacific Ocean, 1992–2021. (**A**) Catch data; (**B**) CPUE data.

Yellowfin tuna is a high-value, widely distributed tropical tuna species, supporting one of the most important industrial fisheries in the western and central Pacific Ocean. In this region, annual catches have exceeded 700,000 metric tonnes in recent years, reflecting both the species’economic importance and the intensity of fishing pressure. To assess the stock’s population dynamics, the model was fitted using annual catch data (Figure 1A) and four independent CPUE indices (Figure 1B), spanning the period 1992–2021, all sourced from the Western and Central Pacific Fisheries Commission (WCPFC).

Four independent CPUE indices derived from longline fleets operating in the western and central Pacific Ocean were used to inform stock abundance dynamics. These indices originate from distant-water fishing nations (DWFNs), the United States, Australia, New Zealand, and Pacific Island countries and territories (PICTs), and are considered independent due to differences in fleet composition and spatial coverage. Each CPUE index was constructed by aggregating catch and effort data from all longline fisheries operating within its respective spatial domain. The indices therefore differ in their geographic extent, fishing strategies, and definitions of fishing effort, providing complementary information on population trends. Each CPUE index assumes proportionality between catch rates and underlying abundance, with potential differences in catchability reflecting fleet- and gear-specific characteristics. Integrating multiple CPUE indices reduces reliance on any single data source and enhances the robustness of abundance estimation.

### 2.2. Model Description

Stock dynamics were assessed using the SPiCT, which integrates catch data and relative abundance indices to estimate population biomass dynamics and management reference points [18]. In SPiCT, exploitable biomass is treated as a latent state variable governed by a generalized surplus production function [19], while stochastic process error accounts for unobserved variability in population dynamics [13]. Observed abundance indices are linked to biomass through index-specific catchability coefficients with lognormal observation error, which allows multiple data sources to be integrated within a unified framework. Model parameters and latent states are estimated efficiently using likelihood-based methods implemented via Template Model Builder (TMB), enabling robust inference and uncertainty quantification for key quantities such as MSY, F_MSY_, and B_MSY_ [20]. Owing to its continuous-time formulation, explicit treatment of uncertainty, and computational efficiency, SPiCT provides a flexible and practical tool for assessing stock status, particularly for data-limited to moderately data-rich fisheries.

In this study, we applied an updated version of the SPiCT model developed by Mildenberger et al. (2025) [21]. The SPiCT model differs from conventional surplus production parameterizations in that population productivity is expressed in terms of maximum net productivity, rather than being parameterized directly by the intrinsic growth rate. Specifically, maximum net productivity is defined as

(1) $$m=\frac{rK}{n^{\frac{n}{n-1}}}$$

where *n* > 0 is a unitless parameter that governs the shape of the surplus production curve [18]. When *n* = 2, the production curve corresponds to the Schaefer surplus production model [22], whereas as n approaches 1 (*n* → 1), it converges to the Fox surplus production model [23]. Within this framework, the biomass dynamics can be further generalized to allow key productivity-related parameters to vary over time. Following the approach of Mildenberger et al. (2020) [24], temporal variation in maximum net productivity (*m_t_*) is permitted, which implies corresponding variation in the intrinsic growth rate ($r_{t}$). In addition, the model formulation allows carrying capacity ($K_{t}$) to vary over time, thereby accommodating longer-term changes in ecosystem productivity or habitat availability. Together, these extensions enable a flexible representation of non-stationary population dynamics within the SPiCT framework:

(2) $$dB_{t}=\left(\gamma m_{t}\frac{B_{t}}{K_{t}}-\gamma m_{t}{\left[\frac{B_{t}}{K_{t}}\right]}^{n}-F_{t}B_{t}\right)dt+\sigma_{B}B_{t}dW_{t}$$

where $\sigma_{B}$ is the standard deviation of the biomass process residuals, $W_{t}$ is standard Brownian motion, and $\gamma ={\frac{n}{n-1}}^{\frac{n}{n-1}}$. In the model, the m at time t is defined as the product of a mean level ($\bar{m}$) and an Ornstein–Uhlenbeck (OU) process [25]: $m_{t}=\bar{m}G_{t}^{\left(m\right)}$. The OU process, $G_{t}^{\left(m\right)}$, is a mean-reverting stochastic process and is described by

(3) $$dlog\left(G_{t}^{\left(m\right)}\right)=-\psi_{m}log\left(G_{t}^{\left(m\right)}\right)dt+\sigma_{m}dW_{t}^{m}$$

where the mean-reversion rate $\psi_{m}$ determines the speed at which the process returns to its long-term mean (here 0), and $\sigma_{m}$ denotes the standard deviation of the process. Similarly, carrying capacity at time t is modeled as a stochastic process around a mean ($\bar{K}$), expressed as the product of the mean level and an OU process, $K_{t}=\bar{K}G_{t}^{\left(K\right)}$, where $G_{t}^{\left(K\right)}$ follows an OU process described by

(4) $$dlog\left(G_{t}^{\left(K\right)}\right)=-\psi_{K}log\left(G_{t}^{\left(K\right)}\right)dt+\sigma_{K}dW_{t}^{K}$$

where $\psi_{K}$ is the rate of mean reversion and $\sigma_{K}$ is the standard deviation of the process. The parameter relationships remain unchanged, such that allowing $m_{t}$ to vary over time induces time variation in the intrinsic growth rate:

(5) $$r_{t}=\frac{m_{t}}{K_{t}}n^{\frac{n}{n-1}}$$

(6) $$G_{t}^{\left(r\right)}=\frac{G_{t}^{\left(m\right)}}{G_{t}^{\left(K\right)}}n^{\frac{n}{n-1}}$$

In this study, the OU process is a stationary Gauss–Markov process interpreted as the continuous-time analog of a first-order auto-regressive model. It characterizes temporally correlated stochastic deviations that gradually revert toward a long-term mean, making it well suited for describing fluctuations around average levels of key biological parameters such as *K_t_* or *m_t_*. The selection of a mean-reverting OU process, rather than a purely non-stationary stochastic process such as a random walk, was motivated by two key considerations. First, the OU formulation limits unbounded parameter drift and helps prevent biologically implausible values (e.g., unrealistically high productivity leading to stock collapse) without requiring overly strong prior constraints, thereby improving numerical stability and ensuring biological realism. Second, because the OU process is fully specified by its fixed effects and stationary variance, the associated random effects follow a well-defined distribution, even during periods with sparse or missing data. This property facilitates model estimation and diagnostic procedures, such as computing one-step-ahead residuals for early observations. In contrast, non-stationary alternatives, such as random walks would require additional constraints, either through explicit bounding or the use of informative priors within a Bayesian framework, to ensure biologically realistic behavior. Given the objectives of this study, the OU process provides a parsimonious and robust representation of time-varying productivity parameters that balances temporal flexibility with biological plausibility [21].

In addition to allowing the productivity-related parameters $m_{t}$ and $K_{t}$ to vary over time, this model also enables the estimation of time-varying biological reference points, which are defined as functions of these parameters according to the following equations:

(7) $$F_{MSY,t}=\frac{m_{t}}{K_{t}{\left(\frac{1}{n}\right)}^{\frac{1}{n-1}}}$$

(8) $$B_{MSY,t}=K_{t}{\left(\frac{1}{n}\right)}^{\frac{1}{n-1}}$$

(9) $${MSY}_{t}=m_{t}$$

The three equations above define the estimation formulas for the time-varying reference points $F_{MSY,t}$, $B_{MSY,t}$, and ${MSY}_{t}$, respectively.

### 2.3. Model Parameterization and Scenarios Design

In addition to the catch and CPUE data used for model fitting, prior information was specified for the model parameters. The priors for the productivity parameters *r* and *K* were specified to follow normal distributions on the log scale, with means equal to the natural logarithms of 0.49 and 11,230,000, and standard deviations of 0.2 and 0.5, respectively. The initial stock status, defined as the ratio of initial biomass to carrying capacity ($B_{0}/K$), was set at 0.46. The prior for the *r* was informed by estimates from a Leslie matrix model [26,27], in which population growth is determined by the dominant eigenvalue of an age-structured projection matrix. The prior for the *K* and the initial exploitation status ($B_{0}/K$) were estimated based on existing assessment outputs for western and central Pacific yellowfin tuna [28].

In this study, three surplus production models were applied: the Schaefer model (with the shape parameter *n* fixed at 2), the Fox model (with *n* fixed as the limiting case as it approaches 1), and the Pella-Tomlinson model (with *n* estimated freely). Furthermore, six alternative scenarios were specified for the different surplus production models, including one static scenario and five time-varying scenarios (Table 1). The four time-varying parameters ($\psi_{m}$, $\sigma_{m}$, $\psi_{K}$, $\sigma_{K}$) were all set to a moderate value of 0.2. The length of the Euler time step was set to 1/8 year.

Because time-varying parameters were incorporated into the model, increasing its overall complexity, the Akaike Information Criterion corrected for small sample sizes (AICc) [29] was used to compare the goodness of fit among different models. Model diagnostics were performed by examining the independence and standard normality of one-step-ahead (OSA) residuals to assess potential violations of model assumptions. Potential violations of the independence assumption were evaluated using the Ljung–Box test [30].

### 2.4. Analysis of Environmental Effects

To investigate the influence of climate-related environmental variability on yellowfin tuna population productivity, we considered a total of ten environmental and large-scale ocean-climate variables that have been widely reported to affect tuna distribution, habitat suitability, and productivity in the tropical western and central Pacific Ocean. The variables comprised sea surface temperature (SST), sea surface salinity (SSS), sea surface dissolved oxygen concentration (SSO), sea surface chlorophyll-a concentration (SSCA), mixed layer thickness (MLT), the El Niño-Southern Oscillation index (ENSO), sea surface height (SSH), the Pacific Decadal Oscillation (PDO), the Interdecadal Pacific Oscillation (IPO), and warm pool sea surface temperature anomalies (WPSTA) (Table 2). Environmental data were obtained from the Copernicus Marine Service (CMS) and the National Oceanic and Atmospheric Administration (NOAA). The dataset covered the entire of the western and central Pacific Ocean and was aggregated to annual means to match the temporal resolution of the fisheries data.

Time-varying estimates of maximum net productivity (*m*), which are numerically equivalent to MSY, derived from surplus production models under different time-varying parameterization scenarios, were used as the response variable in subsequent analyses. Environmental variables were treated as explanatory covariates in the models. A series of GAMs were constructed to evaluate which environmental factors were significantly associated with temporal variation in m (*p* < 0.05) and to characterize the functional form of these relationships. Prior to model fitting, multicollinearity among the environmental variables was assessed using variance inflation factors (VIFs). VIF values were calculated for all candidate environmental variables, and a threshold of 10 was applied to identify instances of problematic multicollinearity. Variables with VIF values exceeding this threshold were excluded from subsequent analyses to reduce redundancy and enhance model interpretability. In addition, year was included as a candidate smooth term in the GAMs to account for potential temporal structure, including long-term trends and low-frequency variability. GAMs were fitted using the mgcv package (version 1.9-1) in R (version 4.4.2). Thin plate regression splines were employed as the smoothing basis for all continuous environmental covariates. The basis dimension (k) for each smooth term was determined based on the sample size and preliminary sensitivity analyses to ensure sufficient model flexibility while avoiding overfitting. Smoothing parameters were estimated using restricted maximum likelihood (REML). Model diagnostics were performed by examining residual patterns and assessing for autocorrelation and systematic deviations. Potential overfitting was evaluated using diagnostic tools implemented in mgcv, including assessments of effective degrees of freedom and the k-index. Model selection was performed using the Akaike Information Criterion (AIC), with lower AIC values indicating a better model fit. For each time-varying scenario, the model with the lowest AIC was selected as the optimal representation of environmental influences on m.

**Figure 2:**
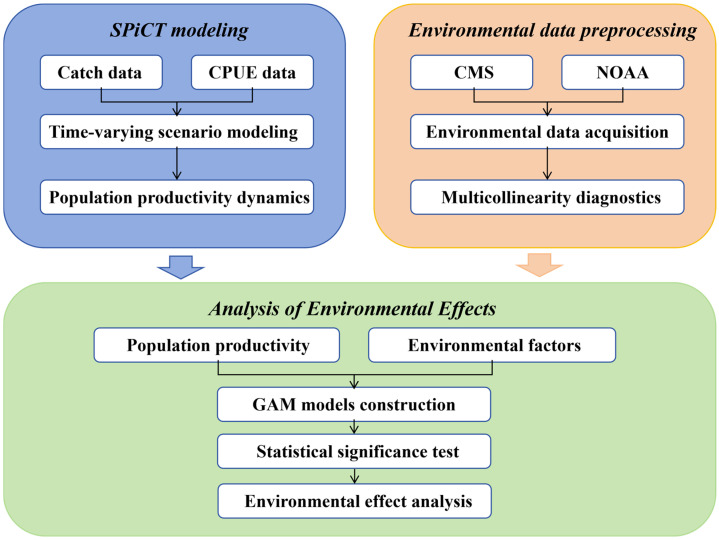
Conceptual framework of the SPiCT modeling and post hoc environmental analysis. Arrows indicate the sequential workflow and analytical steps of the study.

Figure 2 provides a conceptual overview of the modeling framework and analytical workflow. It illustrates the hierarchical structure of the SPiCT state-space model and the subsequent linkage between SPiCT-derived productivity estimates and environmental covariates through a post hoc GAM analysis. The diagram is intended to clarify the overall modeling structure and the sequential relationship between stock assessment and environmental analysis.

## 3. Results

### 3.1. Comparison of Model Fit Across Different Scenarios

Estimates of the parameters *r* and *K*, along with their associated biological reference points varied among surplus production formulations and time-varying scenarios (Table 3). For time-varying models, the reported parameter estimates correspond to values in the terminal assessment year, enabling direct comparison with constant-parameter configurations. Across all surplus production formulations, estimates of r were relatively stable among scenarios, with median values ranging from approximately 0.43 to 0.47 yr^−1^ and overlapping uncertainty intervals. In contrast, estimates of K exhibited greater variation across both model formulations and time-varying scenarios, spanning approximately 5.2–7.8 × 10^6^ t under the Schaefer and Fox models and reaching higher values under the Pella-Tomlinson formulation. Corresponding estimates of MSY also varied across scenarios, with values generally ranging from approximately 0.68 to 1.13 × 10^6^ t, reflecting differences in the underlying productivity and carrying capacity estimates.

Reference points derived from these parameters exhibited consistent patterns across the different surplus production formulations. Estimates of F_MSY_ were formulation-dependent, generally lower values under the Schaefer and Pella-Tomlinson models and higher under the Fox model, while remaining relatively stable across time-varying scenarios within each formulation. Estimates of B_MSY_ exhibited greater variation, particularly in scenarios allowing temporal variation in K, and were generally higher under the Pella-Tomlinson formulation than under the Schaefer and Fox models. Model fit, as assessed by AICc, varied among time-varying scenarios within each surplus production formulation. Across all three formulations, constant or moderately constrained time-varying models tended to achieve lower AICc values than more flexible configurations, whereas scenarios permitting fully independent temporal variation in parameters generally resulted in higher AICc values. Overall, differences in estimated productivity parameters and reference points were jointly influenced by both the choice of surplus production formulation and the assumed time-varying scenario. Estimates of key model parameters, including *r*, *K*, catchability (q), and error terms, were examined to evaluate their plausibility. All parameter estimates fell within biologically and empirically plausible ranges, and none were found to be close to their specified boundary constraints. These results suggest that the model parameters were well identified given the available data. Diagnostic and retrospective analyses for all scenarios are presented in Tables S1 and S2 of the Supplementary Materials, respectively.

**Figure 3:**
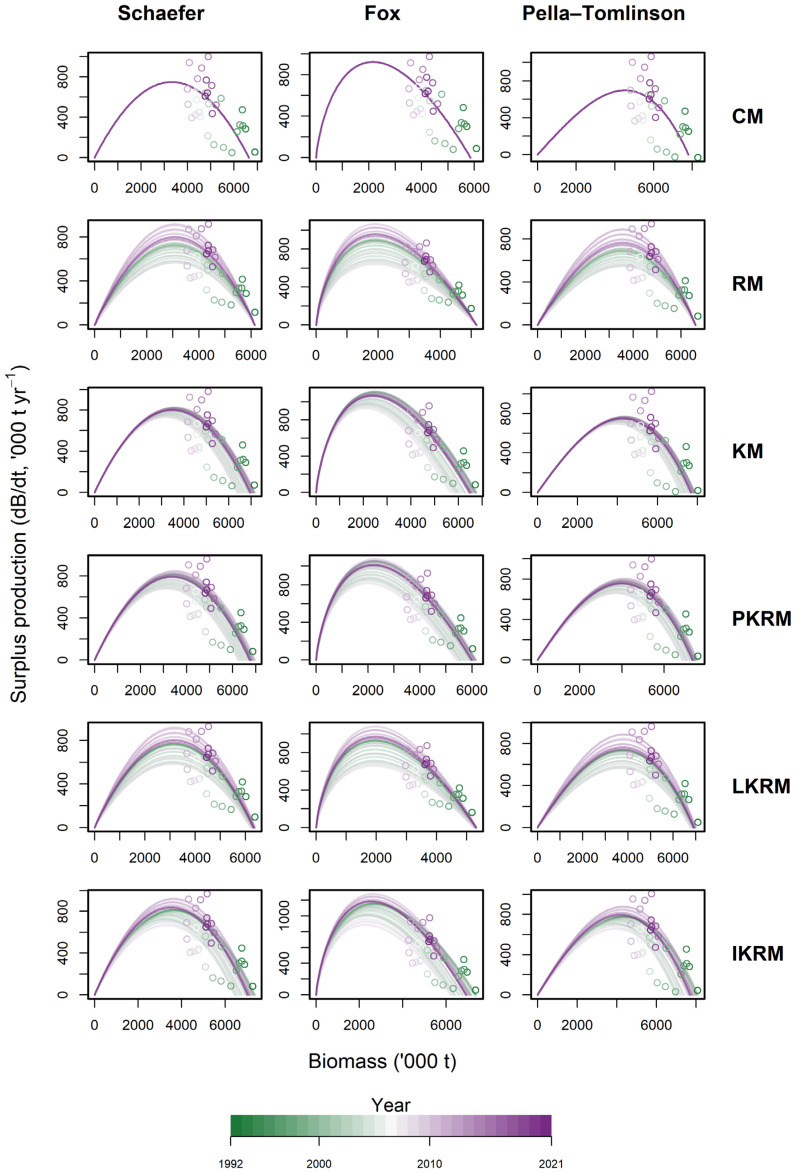
Surplus production curves under alternative production models and time-varying scenarios. Surplus production (dB/dt) as a function of biomass estimated using Schaefer, Fox, and Pella-Tomlinson models across six time-varying scenarios (CM, RM, KM, PKRM, LKRM, and IKRM). Circles represent annual observations, and solid curves indicate estimated production functions, with color gradients denoting years from 1992 to 2021.

Across all scenarios, surplus production exhibited a consistent dome-shaped relationship with biomass, increasing at low biomass levels, peaking at intermediate biomass, and declining at higher biomass (Figure 3). This pattern was observed under the Schaefer, Fox, and Pella-Tomlinson formulations, indicating robust density dependence across different model structures. Distinct differences were evident among the productivity scenarios. Time-varying scenarios (RM, KM, PKRM, LKRM, and IKRM) produced broader envelopes of surplus production curves than the constant-parameter scenario (CM), reflecting interannual variability in productivity and corresponding shifts in the biomass associated with maximum production. In contrast, the CM scenario exhibited a narrower and more static production–biomass relationship. Structural differences among the production models were also apparent. The Fox model generally produced more left-skewed curves with peak production occurring at lower biomass levels, whereas the Schaefer and Pella-Tomlinson models exhibited more symmetric, dome-shaped relationships. Despite these differences, substantial overlap occurred among the models over the biomass range most frequently observed. The distribution of biomass-production observations shifted over time, with earlier observations occurring at higher biomass levels associated with lower surplus production, and more recent observations occurring nearer to the peak of the production curves. Overall, the production–biomass relationships were broadly similar across models, whereas the magnitude and location of surplus production varied with the inclusion of time-varying productivity.

### 3.2. Time-Varying r and K

**Figure 4:**
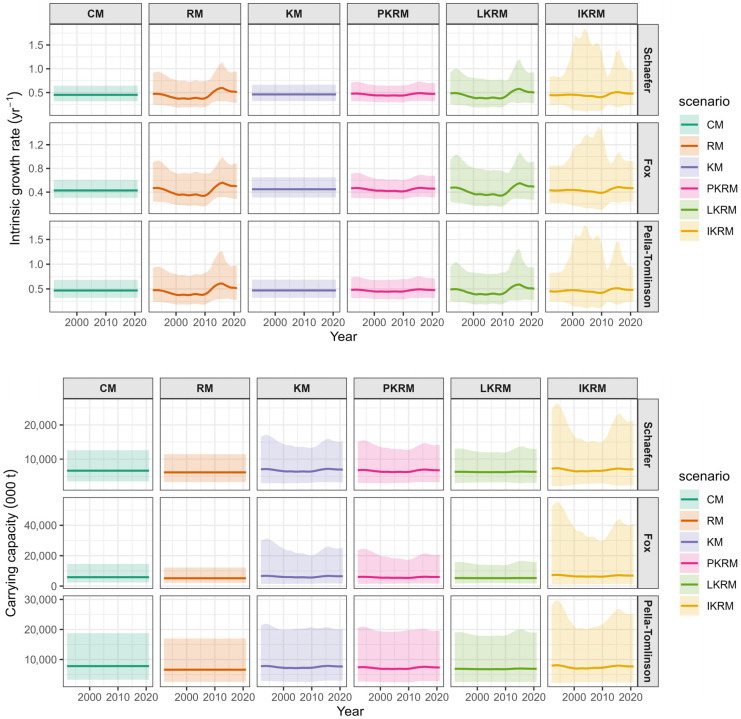
Estimates of time-varying r and K under different scenarios.

Estimates of the *r* and *K* exhibited broadly consistent qualitative patterns across the three surplus production formulations, whereas their magnitude and temporal variability differed substantially among the time-varying scenarios (Figure 4). Under the constant-parameter model (CM), both *r* and *K* remained stable throughout the time series. When only *r* was allowed to vary (RM), *r* exhibited moderate interannual fluctuations, with a decline in the early 2000s followed by an increase in later years, while *K* remained constant throughout the time series. In contrast, when only *K* was allowed to vary (KM), *K* displayed noticeable temporal variation, while *r* remained nearly constant, although *K* estimates were associated with relatively wide uncertainty, particularly under the Pella-Tomlinson formulation. When *r* and *K* were allowed to vary jointly under constrained structures, the proportional model (PKRM) produced highly synchronized and relatively smooth temporal trajectories for both parameters, whereas the power-law coupled model (LKRM) resulted in slightly greater temporal variability while preserving similar overall trends. The fully independent model (IKRM) exhibited the largest temporal fluctuations and the widest uncertainty bands for both parameters. Across formulations, temporal variation was more clearly reflected in *r* than in *K*, except in scenarios where time variation in *K* was explicitly specified.

### 3.3. Time-Varying Biological Reference Points

**Figure 5:**
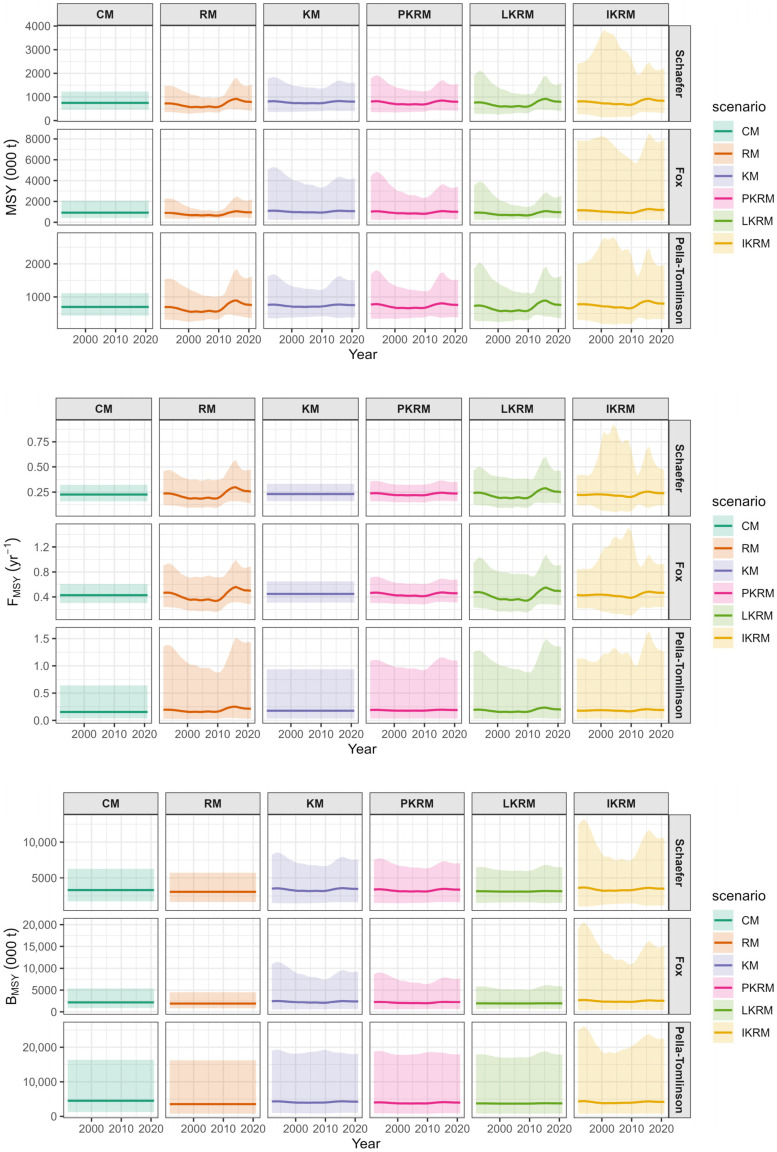
Estimates of time-varying biological reference points (MSY, F_MSY_, and B_MSY_) under different scenarios.

Estimates of the biological reference points (MSY, F_MSY_, and B_MSY_) exhibited broadly consistent qualitative patterns across the three surplus production formulations, whereas their magnitude, temporal variability, and associated uncertainty differed markedly among the time-varying scenarios (Figure 5). Under the constant-parameter model (CM), all three reference points remained stable throughout the time series. When only the r was allowed to vary (RM), moderate interannual variability was observed in MSY, F_MSY_, and B_MSY_, with similar temporal patterns across production functions, including a decline during the early 2000s followed by an increase in more recent years. In contrast, when only the K was allowed to vary (KM), temporal variation was primarily reflected in MSY and B_MSY_, whereas F_MSY_, remained nearly constant throughout the time series. Under this scenario, estimates were generally associated with wider uncertainty bands, particularly for B_MSY_.

When *r* and *K* were allowed to vary jointly under constrained structures, the proportional time-varying model (PKRM) generated relatively smooth and synchronized temporal trajectories for all three reference points. The power-law coupled model (LKRM) exhibited slightly greater temporal variability, while maintaining similar overall trends in MSY, F_MSY_, and B_MSY_. The fully independent time-varying scenario (IKRM) generated the largest apparent interannual fluctuations and the widest uncertainty intervals for all reference points, with estimates exhibiting increased variability across the entire time series. Across all surplus production formulations, temporal variability was most consistently reflected in MSY, followed by B_MSY_, whereas F_MSY_, generally exhibited more limited temporal variation except under the most flexible model structures. The magnitude and temporal dynamics of the biological reference points varied substantially with the assumed time-varying scenario, indicating substantial structural sensitivity in the estimation of time-varying reference points.

### 3.4. Population Status Estimates Under Different Scenarios

**Figure 6:**
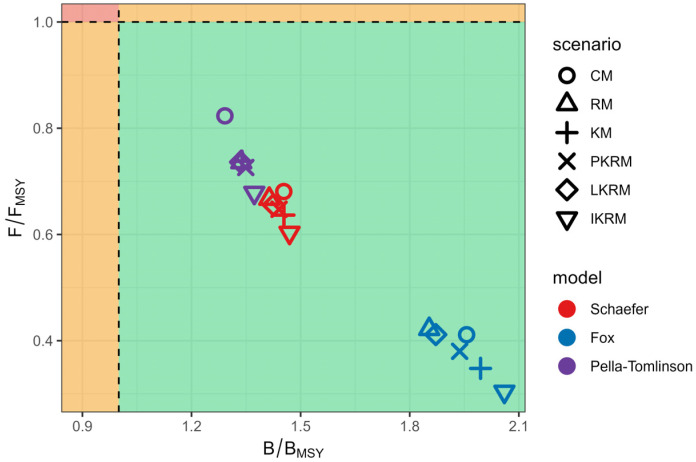
Comparison of stock assessment results across different scenarios. The green region indicates that the stock is in a sustainable state (B/B_MSY_ > 1 and F/F_MSY_ < 1).

Estimated stock status, expressed as B/B_MSY_ and F/F_MSY_, varied among surplus production formulations and time-varying scenarios, but exhibited consistent qualitative patterns across models (Figure 6). Across all formulations and scenarios, estimates fell within the region where B/B_MSY_, greater than one and F/F_MSY_, less than one. Under the Schaefer formulation, stock status estimates clustered within a relatively narrow range, with moderate B/B_MSY_ and F/F_MSY_ values and showed limited differences among time-varying scenarios. The Fox formulation produced higher B/B_MSY_ estimates and lower F/F_MSY_ values, with greater dispersion across scenarios, particularly under more flexible time-varying structures. Estimates under the Pella-Tomlinson formulation were characterized by the lowest B/B_MSY_ and the highest F/F_MSY_ values among the three surplus production formulations. Variation in estimated stock status was more strongly associated with the choice of surplus production formulation than with the specific time-varying scenario, whereas the scenario effects primarily influenced the relative position of estimates within each formulation.

### 3.5. Correlation Analysis of Environmental Factors

Multicollinearity among the ten candidate environmental variables was assessed using variance inflation factors (VIFs). The results showed that sea surface temperature (SST), sea surface height (SSH), and the El Niño–Southern Oscillation (ENSO) index exhibited strong multicollinearity with other variables and were therefore excluded (Table 4). The remaining environmental variables were retained for subsequent generalized additive model (GAM) analyses.

The optimal GAM formulations varied among the time-varying scenarios, but consistently included a smooth temporal term along with a subset of environmental covariates (Table 5). Across all scenarios, the PDO was retained in the optimal models and exhibited a statistically significant association with *m*, with *p*-values ranging from 0.001 to 0.007. MLT was likewise selected in all optimal models, although its statistical significance varied among scenarios. MLT was highly significant in the KM, PKRM, and LKRM scenarios (*p* ≤ 0.004), marginally significant in the IKRM scenario (*p* = 0.049), and not-significant in the RM scenario (*p* = 0.055). SSS was included the optimal models for the KM, PKRM, LKRM, and IKRM scenarios; however, its effects were not statistically significant in any case (*p* > 0.05). In contrast, SSO was only retained in the optimal model under the RM scenario and did not exhibit a statistically significant association with m (*p* = 0.674). In summary, the results indicate that PDO is the most consistently significant environmental factor across the time-varying scenarios, whereas MLT also shows statistically significant associations with yellowfin tuna population productivity, although its relative importance varies among scenarios.

**Figure 7:**
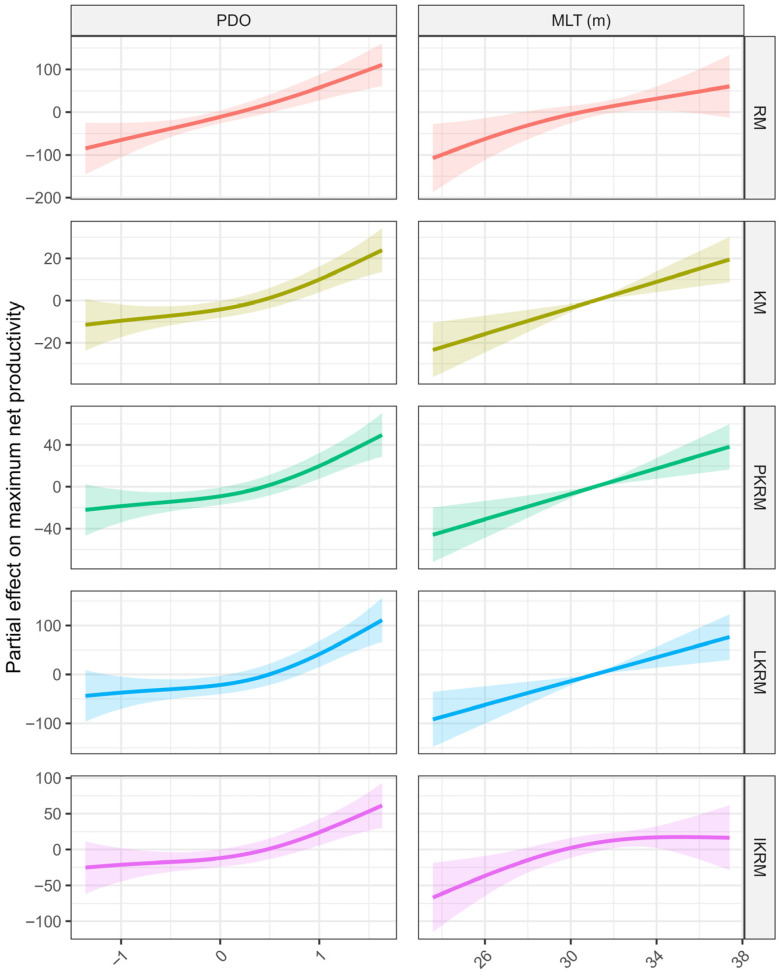
Environmental effects on maximum net productivity across time-varying model scenarios. Different colored lines represent different time-varying scenarios.

Generalized additive model results revealed broadly consistent patterns in the relationships between environmental variables and maximum net productivity across time-varying scenarios, although the magnitude and shape of the responses varied among scenarios (Figure 7). The PDO showed a consistently positive association with net productivity in all scenarios where it was retained. Across the RM, KM, PKRM, LKRM, and IKRM scenarios, net productivity increased progressively with higher PDO values, with relatively narrow confidence intervals over most of the observed range, indicating a stable and robust relationship. MLT likewise exhibited a generally positive effect on net productivity across the scenarios. Increases in MLT were associated with higher net productivity across all time-varying models, although the response curves differed slightly in curvature and associated uncertainty. In several scenarios, the effect of MLT appeared approximately linear across the central range of observations, while increased uncertainty was observed at the lower and upper extremes.

## 4. Discussion

The contrasting sensitivities of *r* and *K* estimates to model structure and time-varying assumptions are consistent with findings from previous surplus production and state-space assessments. The relative stability of *r* across different formulations and scenarios likely reflects its strong identifiability from population growth dynamics at low biomass, a pattern commonly reported in surplus production models [4,13]. In contrast, the greater variation observed in *K* estimates highlights their dependence on model formulation and assumptions about temporal variability, particularly in models that allow *K* to vary over time. This sensitivity of *K* has been observed in both simulation and empirical studies, where changes in environmental conditions or underlying productivity states can be absorbed into *K* rather than *r*, leading to divergent estimates across model structures [5,12]. Furthermore, the tendency of constant or moderately constrained time-varying models to achieve lower AICc values than more flexible configurations is consistent with the effects of over-parameterization when data are insufficient to support highly flexible temporal dynamics [31]. Together, these results underscore the importance of carefully balancing biological realism and model parsimony when incorporating time-varying processes into surplus production assessments.

The consistently dome-shaped surplus production–biomass relationships observed across production models align with classical surplus production theory, which posits density-dependent regulation of population growth and declining net production at high biomass levels [22,32]. The persistence of this pattern across the Schaefer, Fox, and Pella-Tomlinson formulations indicates that the overall production–biomass structure is robust to alternative functional forms, although differences in curve shape reflect distinct assumptions regarding stock dynamics. In particular, the left-skewed production curves generated by the Fox model are a well-known consequence of its exponential formulation, implying relatively higher productivity at lower biomass compared with logistic-based models [23,33]. The broader envelopes of surplus production curves under time-varying scenarios suggest that permitting productivity-related parameters to vary through time captures interannual variability that is not represented under constant-parameter assumptions. Similar effects have been reported in both empirical assessments and simulation studies, where temporal variation in productivity leads to shifting production–biomass relationships and increased uncertainty around reference points [34,35]. The shift from higher biomass and lower surplus production in earlier years toward conditions closer to the production peak in more recent years underscores the importance of accounting for time-varying productivity when interpreting recent stock dynamics and production potential.

Our results showed that *r* and *K* exhibited broadly consistent overall patterns across surplus production formulations, suggesting that the data contain sufficient information to identify general productivity dynamics, while substantial differences in magnitude and temporal variability among time-varying scenarios reflect strong sensitivity to structural assumptions. The relative stability of *r* and *K* under the constant-parameter model is expected, because temporal variation is effectively absorbed into process and observation errors when parameters are held fixed. Allowing only *r* to vary captured moderate interannual fluctuations in the population growth rate, a pattern commonly observed in surplus production and state-space models in which *r* is more directly informed by short-term changes in biomass dynamics [36,37]. In contrast, temporal variation in *K* was associated with greater uncertainty, particularly under more flexible formulations, consistent with previous findings that *K* is often weakly identifiable and highly sensitive to model structure and parameterization [38,39].

Differences among jointly varying scenarios further illustrate the influence of structural constraints. The smooth and highly synchronized trajectories produced by the PKRM indicate that coupling *r* and *K* can effectively regularize time-varying dynamics, whereas the increased variability under the LKRM and especially the IKRM reflects the relaxation of these constraints. Similar trade-offs between model flexibility and uncertainty have been reported in simulation and empirical studies, where highly flexible time-varying models tend to exhibit wider uncertainty bands unless strongly supported by data [21,31]. Taken together, inferred temporal dynamics in productivity parameters depend as much on structural assumptions as on the underlying data, emphasizing the need for carefully chosen constraints when modeling time-varying processes in surplus production assessments. The differing uncertainty patterns observed across scenarios partly reflect the identifiability limitations of time-varying *r* and *K*. When catch and CPUE provide limited information, *r* and *K* can exhibit negative correlation, with increases in one parameter partially compensated by decreases in the other. This parameter confounding contributes to higher uncertainty in productivity estimates during certain periods, highlighting the importance of interpreting model outputs in the context of underlying data limitations.

The fact that stock status estimates consistently fall within the region defined by B/B_MSY_ > 1 and F/F_MSY_ < 1 across all formulations and scenarios suggests broad agreement regarding the overall relative stock condition, despite differences in model structure and time-varying assumptions. Simultaneously, the formulation-dependent differences in B/B_MSY_ and F/F_MSY_ highlight the substantial influence of surplus production model structure on stock status metrics derived from model-based reference points. Higher B/B_MSY_ and lower F/F_MSY_ estimates under the Fox formulation are consistent with its exponential production function, which implies lower biomass at maximum production and relatively higher productivity at low stock sizes compared with logistic-based models [23,40]. In contrast, the lower B/B_MSY_ and higher F/F_MSY_ values obtained under the Pella-Tomlinson formulation reflect the sensitivity of reference points and stock status to assumptions regarding the curvature of the production function. The comparatively smaller differences among time-varying scenarios within each formulation suggest that stock status estimates are more strongly influenced by the choice of surplus production formulation than by the specific representation of time-varying productivity. A similar dominance of model structure over alternative parameterizations has been reported in comparative assessments, where different surplus production models fitted to the same data can lead to systematically different estimates of stock status [41]. Nevertheless, the greater dispersion observed under more flexible time-varying scenarios suggests that time-varying processes can influence the relative positioning of estimates within a given formulation, underscoring the importance of considering both structural and temporal sources of uncertainty when interpreting stock status indicators.

Our results indicate that the PDO and MLT significant influence on the population productivity of yellowfin tuna, with consistent effects observed across alternative time-varying scenarios. The strong positive association between PDO and maximum net productivity suggests that basin-scale climate variability plays a central role in modulating productivity through its effects on the pelagic ecosystem. Positive phases of the PDO are linked to large-scale changes in sea surface temperature, upper-ocean stratification, and nutrient distributions across the Pacific basin, which can alter primary productivity and the spatial structure of prey fields in the western and central Pacific [42,43]. For highly mobile pelagic predators such as yellowfin tuna, these climate-driven ecosystem changes can improve feeding conditions, enhance growth, and increase recruitment success, thereby helping to explain the observed rise in net productivity under higher PDO values. Beyond its effects on lower-trophic production, PDO-related variability may influence the horizontal and vertical distribution of prey species, thereby modifying habitat suitability and foraging efficiency for yellowfin tuna [44,45]. Basin-scale shifts in oceanographic conditions can reshape frontal systems, convergence zones, and mesoscale features that structure pelagic food webs, potentially leading to more favorable spatial overlap between predators and prey during positive PDO phases [46]. Such indirect ecosystem-mediated pathways are consistent with previous ecosystem-based modeling studies that link large-scale climate variability to tuna productivity via changes in prey availability and habitat structure [43].

The positive relationship between MLT and net productivity is also mechanistically plausible as mixed-layer depth strongly regulates light availability, vertical mixing, and nutrient entrainment, thereby affecting primary production and the transfer of energy to higher trophic levels [47,48]. In tropical and subtropical oceans, deeper mixed-layer periods can enhance nutrient supply from below the euphotic zone through entrainment and mixing, supporting higher phytoplankton production and, ultimately, greater prey availability for tuna and other pelagic predators, particularly under nutrient-limited conditions [43,49]. For highly mobile predators such as yellowfin tuna, increased prey availability within the upper mixed layer can enhance feeding efficiency and growth, while also affecting habitat use and aggregation patterns [50]. Enhanced mixing may also increase the vertical overlap between predators and prey by redistributing zooplankton and micronekton within depths accessible to surface-oriented tunas, thereby strengthening trophic transfer efficiency [51]. Collectively, these processes help explain how changes in mixed-layer structure can lead to differences in population-level productivity, even when tuna exhibit relatively weak direct physiological responses to environmental variability.

Environmental effects on population productivity may operate with temporal lags, such as through delayed responses in recruitment or food-web processes. In this study, we focused on contemporaneous associations between environmental variables and productivity for parsimony and due to data limitations. Future work could explicitly explore lagged environmental effects to further elucidate the mechanistic linkages between climate variability and population dynamics. Our results indicate that temporal structure plays an important role in explaining variability in the response variable. The smooth term for year was not imposed a priori but was consistently retained during the model selection process, suggesting the presence of temporal structure that is not fully captured by the selected environmental covariates. We recognize that this temporal component may partly overlap with low-frequency variability associated with large-scale climate indices. Nevertheless, the year term is interpreted as reflecting residual or cumulative temporal effects, rather than representing a direct mechanistic driver. By accounting for this background temporal structure, the modeling framework reduces the risk of attributing long-term trends to individual environmental variables.

From a management perspective, the use of time-varying reference points fundamentally alters the interpretation of conventional stock status indicators such as B/B_MSY_ and F/F_MSY_. Our results suggest that deviations from these ratios may partly reflect environmentally driven changes in productivity rather than changes in fishing pressure alone. For management bodies such as the WCPFC, time-varying reference points could therefore be used as complementary information to existing assessment outputs, helping to contextualize stock status and distinguish between fishing- and environment-driven dynamics. While the operational implementation of fully dynamic reference points remains challenging, incorporating productivity variability into management advice could improve risk-based decision-making and support more climate-resilient fisheries management.

Several limitations should be acknowledged in this study. First, although surplus production models provide a tractable framework for exploring time-varying productivity, they remain simplified representations of population dynamics and do not explicitly resolve age structure, species interactions, or spatial heterogeneity. As a result, some ecological processes influencing yellowfin tuna productivity may be implicitly absorbed into time-varying parameters rather than being explicitly modeled. Moreover, productivity estimates are derived at the basin scale, which may mask substantial spatial heterogeneity across the expansive western and central Pacific Ocean, where environmental conditions, stock structure, and fishing pressure vary markedly among regions. Second, the identification of environmental effects using GAMs is constrained by data availability and collinearity among covariates, particularly at the extremes of environmental gradients, which introduces uncertainty in the estimated relationships. In addition, changes in estimated productivity may reflect a combination of environmentally driven variability and fishing-induced changes in population structure, which are difficult to fully disentangle within a surplus production modeling framework. Third, CPUE-based abundance indices are sensitive to the choice of standardization methods, and alternative modeling assumptions could influence inferred temporal patterns and, consequently, estimates of time-varying productivity. Finally, the analysis focuses on a limited set of large-scale and upper-ocean environmental indicators and does not account for potential lagged effects or nonlinear interactions among drivers. Future work could address these limitations by integrating spatially explicit or age-structured assessment frameworks, as well as ecosystem-based models, to better resolve the mechanisms linking climate variability, food-web dynamics, and tuna productivity. Incorporating additional environmental variables, exploring lagged and cumulative effects, and evaluating model performance under alternative climate scenarios would further improve understanding of productivity dynamics and strengthen the application of time-varying approaches for ecosystem-based fisheries management.

## 5. Conclusions

This study employs surplus production models with time-varying parameters to evaluate yellowfin tuna productivity in the western and central Pacific Ocean and to investigate the influence of environmental variability. Across alternative time-varying scenarios, model results consistently demonstrate that environmental variability contributes substantially to temporal variation in population productivity. In particular, the PDO and MLT exhibited statistically significant and consistent associations with maximum net productivity. Higher PDO values were associated with increased productivity, whereas greater mixed layer thickness was generally linked to higher production potential, reflecting the sensitivity of yellowfin tuna productivity to basin-scale climate variability and upper-ocean conditions. These findings suggest that incorporating key environmental indicators into surplus production assessments can enhance interpretation of productivity dynamics without substantially increasing model complexity. More broadly, the results underscore the relevance of ecosystem-informed approaches for understanding and managing pelagic fish stocks under a changing climate and highlight the potential value of integrating environmental information into fisheries assessment and management frameworks.

## Figures and Tables

**Table 1:** Scenario settings including one static and five time-varying scenarios.

Scenario	Details	*r*	*K*	Time-Varying Parameters	Dependency
Constant-parameter model (CM)	Both the K and the r were treated as constant through time.	$G_{t}^{\left(r\right)}=1$	$G_{t}^{\left(K\right)}=1$		
Time-varying r model (RM)	The r was allowed to vary over time, whereas the K was assumed to be constant.	$G_{t}^{\left(r\right)}\neq 1$	$G_{t}^{\left(K\right)}=1$	$\psi_{m}$, $\sigma_{m}$	
Time-varying K model (KM)	The K was allowed to vary over time, whereas the r was assumed to be constant.	$G_{t}^{\left(r\right)}=1$	$G_{t}^{\left(K\right)}\neq 1$	$\psi_{K}$, $\sigma_{K}$	
Proportional K and r covarying model (PKRM)	Proportional K and r covarying model. The K and r were assumed to vary proportionally through time.	$G_{t}^{\left(r\right)}\neq 1$	$G_{t}^{\left(K\right)}\neq 1$	$\psi_{m}$, $\sigma_{m}$, $\psi_{K}$, $\sigma_{K}$	$G_{t}^{\left(r\right)}=G_{t}^{\left(K\right)}n^{\frac{n}{n-1}}$
Linked K and r covarying model (Power-law form) (LKRM)	The K and r rate were assumed to co-vary over time according to a power-law relationship with exponent λ.	$G_{t}^{\left(r\right)}\neq 1$	$G_{t}^{\left(K\right)}\neq 1$	$\psi_{m}$, $\sigma_{m}$, $\psi_{K}$, $\sigma_{K}$	$G_{t}^{\left(r\right)}={\left(G_{t}^{\left(K\right)}\right)}^{\lambda }n^{\frac{n}{n-1}}$
Independently varying K and r model (IKRM)	The K and r varied independently over time.	$G_{t}^{\left(r\right)}\neq 1$	$G_{t}^{\left(K\right)}\neq 1$	$\psi_{m}$, $\sigma_{m}$, $\psi_{K}$, $\sigma_{K}$	Independent

**Table 2:** Environmental and climate variables used in the analysis.

Variable	Abbreviation	Data Source	Spatial Resolution	Temporal Aggregation	Definition
Sea Surface Temperature	SST	Copernicus Marine Service	0.25° × 0.25°	Annual mean (1992–2021)	Sea surface temperature, representing the thermal conditions of the ocean surface layer.
Sea Surface Salinity	SSS				Sea surface salinity, reflecting surface water mass properties and ocean circulation.
Sea Surface Dissolved Oxygen Concentration	SSO				Dissolved oxygen concentration at the sea surface, indicating oxygen-related habitat conditions.
Sea Surface Chlorophyll-a Concentration	SSCA				Chlorophyll-a concentration at the sea surface, commonly used as a proxy for primary productivity.
Mixed Layer Thickness	MLT				Thickness of the ocean surface mixed layer, associated with vertical mixing and upper-ocean structure.
Sea Surface Height	SSH				Sea surface height relative to the geoid, reflecting large-scale circulation and mesoscale ocean dynamics.
El Niño–Southern Oscillation	ENSO	National Oceanic and Atmospheric Administration	Basin-scale index		A large-scale climate mode in the tropical Pacific, represented by the Niño 3.4 sea surface temperature index.
Pacific Decadal Oscillation	PDO				A basin-scale climate index describing decadal variability in North Pacific sea surface temperature patterns.
Interdecadal Pacific Oscillation	IPO				A low-frequency climate mode representing interdecadal variability in Pacific basin sea surface temperature.
Warm Pool Sea Surface Temperature Anomalies	WPSTA				Sea surface temperature anomalies averaged over the western Pacific warm pool region, calculated relative to a long-term climatological mean.

**Table 3:** Comparison of model fit and estimates of productivity parameters and biological reference points across scenarios (for time-varying models, estimates are reported for the terminal year, 2021; values in parentheses indicate 95% confidence intervals).

**Schaefer**						
Parameter	CM	RM	KM	PKRM	LKRM	IKRM
r	0.45 (0.32–0.64)	0.46 (0.32–0.67)	0.46 (0.32–0.66)	0.46 (0.32–0.67)	0.46 (0.32–0.67)	0.46 (0.32–0.67)
K (×10^3^ t)	6633 (3499–12,573)	6143 (3290–11,469)	6802 (3145–14,706)	6608 (3150–13,861)	6285 (3114–12,684)	6909 (2408–19,823)
*MSY* (×10^3^ t)	739 (458–1227)	710 (426–1182)	784 (397–1550)	763 (405–1439)	725 (401–1308)	800 (286–2234)
*F_MSY_*	0.23 (0.16–0.32)	0.23 (0.16–0.33)	0.23 (0.16–0.33)	0.23 (0.16–0.33)	0.23 (0.16–0.33)	0.23 (0.16–0.33)
*B_MSY_* (×10^3^ t)	3317 (1750–6287)	3072 (1645–5735)	3401 (1573–7353)	3304 (1575–6931)	3142 (1557–6342)	3454 (1204–9912)
AICc	−53.44	−49.17	−48.76	−48.91	−46.70	−43.79
**Fox**						
Parameter	CM	RM	KM	PKRM	LKRM	IKRM
r	0.43 (0.30–0.61)	0.45 (0.31–0.65)	0.45 (0.31–0.65)	0.45 (0.31–0.65)	0.45 (0.31–0.65)	0.45 (0.31–0.67)
K (×10^3^ t)	5852 (2346–14,602)	5164 (2178–12,245)	6300 (1593–24,909)	5834 (1651–20,612)	5286 (1823–15,330)	6782 (1126–40,847)
*MSY* (×10^3^ t)	922 (409–2079)	851 (388–1869)	1039 (258–4184)	959 (278–3304)	870 (323–2346)	1128 (169–7526)
*F_MSY_*	0.43 (0.30–0.61)	0.45 (0.31–0.65)	0.45 (0.31–0.65)	0.45 (0.31–0.64)	0.45 (0.31–0.65)	0.45 (0.31–0.67)
*B_MSY_* (×10^3^ t)	2154 (863–5374)	1901 (802–4507)	2319 (586–9168)	2147 (608–7587)	1946 (671–5642)	2496 (414–15,034)
AICc	−52.41	−48.70	−48.26	−48.41	−46.20	−43.35
**Pella-Tomlinson**						
Parameter	CM	RM	KM	PKRM	LKRM	IKRM
r	0.47 (0.32–0.68)	0.47 (0.32–0.68)	0.47 (0.32–0.69)	0.47 (0.32–0.69)	0.47 (0.32–0.68)	0.47 (0.32–0.69)
K (×10^3^ t)	7802 (3246–18,754)	6604 (2566–16,998)	7542 (2834–20,071)	7215 (2655–19,611)	6869 (2531–18,637)	7574 (2351–24,403)
*MSY* (×10^3^ t)	697 (436–1115)	683 (378–1234)	739 (387–1412)	731 (391–1365)	693 (369–1302)	768 (326–1812)
*F_MSY_*	0.15 (0.04–0.64)	0.19 (0.03–1.16)	0.18 (0.03–0.94)	0.19 (0.03–1.04)	0.19 (0.03–1.11)	0.19 (0.03–1.07)
*B_MSY_* (×10^3^ t)	4520 (1249–16,358)	3547 (776–16,222)	4181 (959–18,228)	3925 (852–18,086)	3737 (793–17,604)	4124 (774–21,970)
AICc	−54.48	−49.92	−49.56	−49.68	−47.43	−44.48

**Table 4:** Results of multicollinearity diagnostics using variance inflation factors (VIFs). Each row represents one step of the iterative screening process, in which the variable with the highest VIF was sequentially removed until all remaining variables had VIF values below 10.

	Environmental Variables									
	SST	SSS	SSO	SSCA	SSH	ENSO	MLT	PDO	IPO	WPSTA
Variance inflation factors	125.91	31.99	24.47	9.47	79.92	102.78	33.17	17.02	49.18	39.72
		7.27	22.69	9.23	26.61	47.17	9.61	15.03	36.65	14.73
		5.08	9.32	5.13	24.45		8.70	5.32	10.50	6.42
		3.92	8.61	4.28			3.48	4.89	4.72	6.31

**Table 5:** Optimal GAM fitting results for each time-varying scenario.

Scenario	Optimal Model Formulation	*p*-Value			
		PDO	MLT	SSS	SSO
RM	m ~ s(year, k = 3) + s(PDO) + s(MLT, k = 4) + s(SSO)	0.002	0.055		0.674
KM	m ~ s(year, k = 3) + s(PDO) + s(MLT) + s(SSS)	0.001	0.002	0.061	
PKRM	m ~ s(year, k = 3) + s(PDO) + s(MLT) + s(SSS)	0.001	0.002	0.066	
LKRM	m ~ s(year, k = 3) + s(PDO) + s(MLT, k = 3) + s(SSS)	0.001	0.004	0.062	
IKRM	m ~ s(year, k = 3) + s(PDO) + s(MLT, k = 3) + s(SSS)	0.007	0.049	0.182	

## Data Availability

The original contributions presented in this study are included in the article/Supplementary Materials. Further inquiries can be directed to the corresponding authors.

## Supplementary Materials

The following supporting information can be downloaded at https://www.mdpi.com/article/10.3390/ani16050856/s1, Table S1: Model diagnostic results for different scenarios (*p* ≥ 0.05 indicates no statistically significant evidence of violations of model assumptions). The OSA bias test assesses systematic bias in the residuals; the ACF Ljung–Box test evaluates temporal autocorrelation in the residuals; and the Shapiro–Wilk test examines the normality of the residuals; Table S2: Retrospective analysis results under different scenarios (Mohn’s ρ values between −0.2 and 0.2 indicate that retrospective bias is within an acceptable range).
