# Supplementary material for: Climate-Driven Variation in Yellowfin Tuna Productivity in the Western and Central Pacific Ocean Inferred from a State-Space Model
Source: Animals (Basel). 2026 Mar 9;16(5):856. doi: 10.3390/ani16050856 (PMC12984235; doi:10.3390/ani16050856)
Supplement: Supplementary file 1 [file animals-16-00856-s001.zip › animals-4145044-supplementary.pdf]

Climate-Driven Variation in Yellowfin Tuna Productivity in the Western and Central Pacific Ocean Inferred from a State-Space Model

Supplementary Materials

Table S1. Model diagnostic results for different scenarios ($p \geq 0.05$ indicates no statistically significant evidence of violations of model assumptions). The OSA bias test assesses systematic bias in the residuals; the ACF Ljung–Box test evaluates temporal autocorrelation in the residuals; and the Shapiro–Wilk test examines the normality of the residuals.

		OSA bias test					ACF Ljung–Box test					Shapiro–Wilk test				
Model	Scenario	catch	Index1	Index2	Index3	Index4	catch	Index1	Index2	Index3	Index4	catch	Index1	Index2	Index3	Index4
Schaefer	CM	0.05	0.74	0.82	0.56	0.69	0.09	0.06	0.08	0.13	0.28	0.77	0.78	0.29	0.22	0.41
	RM	0.05	0.75	0.82	0.55	0.70	0.07	0.09	0.13	0.13	0.25	0.83	0.97	0.32	0.28	0.54
	KM	0.06	0.69	0.77	0.53	0.66	0.06	0.08	0.09	0.13	0.28	0.73	0.80	0.30	0.27	0.54
	PKRM	0.06	0.69	0.77	0.53	0.66	0.06	0.09	0.10	0.13	0.27	0.72	0.85	0.33	0.28	0.54
	LKRM	0.06	0.73	0.80	0.54	0.69	0.07	0.09	0.12	0.13	0.25	0.81	0.96	0.33	0.28	0.53
	IKRM	0.06	0.70	0.78	0.53	0.66	0.06	0.10	0.10	0.13	0.27	0.75	0.85	0.34	0.29	0.58
Fox	CM	0.05	0.75	0.83	0.56	0.69	0.10	0.06	0.07	0.12	0.30	0.85	0.77	0.31	0.17	0.35
	RM	0.07	0.70	0.78	0.53	0.67	0.07	0.09	0.12	0.13	0.25	0.79	0.95	0.31	0.28	0.53
	KM	0.07	0.66	0.76	0.52	0.64	0.06	0.11	0.09	0.13	0.28	0.72	0.80	0.33	0.24	0.54
	PKRM	0.07	0.66	0.75	0.52	0.64	0.07	0.10	0.10	0.13	0.27	0.72	0.83	0.34	0.25	0.53

	LKRM	0.07	0.69	0.77	0.53	0.66	0.07	0.09	0.12	0.13	0.25	0.78	0.94	0.32	0.27	0.52
	IKRM	0.07	0.67	0.76	0.52	0.65	0.06	0.12	0.10	0.14	0.27	0.75	0.85	0.36	0.25	0.56
Pella-Tomlinson	CM	0.04	0.75	0.82	0.56	0.70	0.07	0.06	0.09	0.13	0.27	0.70	0.87	0.30	0.24	0.43
	RM	0.04	0.77	0.83	0.56	0.71	0.07	0.09	0.13	0.13	0.25	0.84	0.98	0.32	0.28	0.54
	KM	0.06	0.70	0.78	0.53	0.67	0.06	0.08	0.09	0.13	0.27	0.73	0.83	0.31	0.27	0.52
	PKRM	0.06	0.70	0.78	0.53	0.67	0.06	0.08	0.10	0.13	0.27	0.72	0.87	0.33	0.28	0.53
	LKRM	0.05	0.74	0.81	0.55	0.70	0.07	0.09	0.12	0.13	0.25	0.82	0.96	0.34	0.29	0.53
	IKRM	0.06	0.70	0.79	0.53	0.67	0.06	0.10	0.10	0.13	0.27	0.75	0.86	0.33	0.30	0.59

Table S2. Retrospective analysis results under different scenarios (Mohn's ρ values between −0.2 and 0.2 indicate that retrospective bias is within an acceptable range).

Model	Scenario	Mohn's ρ F/Fmsy	Mohn's ρ B/Bmsy
Schaefer	CM	0.013	0.002
	RM	-0.032	0.016
	KM	0.005	0.011
	PKRM	-0.013	0.012
	LKRM	-0.052	0.022
	IKRM	0.141	0.028
Fox	CM	0.068	-0.013
	RM	-0.060	0.027
	KM	-0.018	0.012
	PKRM	-0.035	0.018
	LKRM	0.010	0.009

	IKRM	0.030	0.004
	CM	-0.005	0.008
	RM	-0.002	-0.004
Pella-Tomlinson	KM	0.031	-0.001
	PKRM	0.003	0.004
	LKRM	-0.017	0.001
	IKRM	0.095	-0.009
